# Meningitis Outbreak Caused by Vaccine-Preventable Bacterial Pathogens — Northern Ghana, 2016

**DOI:** 10.15585/mmwr.mm6630a2

**Published:** 2017-08-04

**Authors:** Fortress Y. Aku, Fernanda C. Lessa, Franklin Asiedu-Bekoe, Phoebe Balagumyetime, Winfred Ofosu, Jennifer Farrar, Mahamoudou Ouattara, Jeni T. Vuong, Kofi Issah, Joseph Opare, Sally-Ann Ohene, Charles Okot, Ernest Kenu, Donne K. Ameme, David Opare, Abass Abdul-Karim

**Affiliations:** ^1^Ghana Field Epidemiology and Laboratory Training Programme, School of Public Health, University of Ghana, Accra, Ghana; ^2^Ghana Health Service, Accra, Ghana; ^3^Division of Bacterial Diseases, National Center for Immunization and Respiratory Diseases, CDC; ^4^Jirapa Health District, Jirapa, Ghana; ^5^Upper West Regional Directorate, Wa, Ghana; ^6^Upper East Regional Directorate, Bolgatanga, Ghana; ^7^World Health Organization Country Office Ghana, Accra, Ghana; ^8^National Public Health Reference Laboratory, Ghana Health Service, Accra, Ghana; ^9^Zonal Public Health Laboratory, Tamale, Ghana.

Bacterial meningitis is a severe, acute infection of the fluid surrounding the brain and spinal cord that can rapidly lead to death. Even with recommended antibiotic treatment, up to 25% of infected persons in Africa might experience neurologic sequelae (*1*). Three regions in northern Ghana (Upper East, Northern, and Upper West), located in the sub-Saharan “meningitis belt” that extends from Senegal to Ethiopia, experienced periodic outbreaks of meningitis before introduction of serogroup A meningococcal conjugate vaccine (MenAfriVac) in 2012 (*2*,*3*). During December 9, 2015–February 16, 2016, a total of 432 suspected meningitis cases were reported to health authorities in these three regions. The Ghana Ministry of Health, with assistance from CDC and other partners, tested cerebrospinal fluid (CSF) specimens from 286 patients. In the first 4 weeks of the outbreak, a high percentage of cases were caused by *Streptococcus pneumoniae*; followed by an increase in cases caused by *Neisseria meningitidis*, predominantly serogroup W. These data facilitated Ghana’s request to the International Coordinating Group* for meningococcal polysaccharide ACW vaccine, which was delivered to persons in the most affected districts. Rapid identification of the etiologic agent causing meningitis outbreaks is critical to inform targeted public health and clinical interventions, including vaccination, clinical management, and contact precautions.

On December 9, 2015, a patient was evaluated at a hospital in the Savelugu-Nantom district of the Northern Region for fever, headache, vomiting, and neck stiffness. By December 31, 2015, five more patients in the Northern Region and 11 in the Upper West Region were hospitalized with similar symptoms. After ruling out malaria, hospital personnel suspected meningitis and alerted district and regional health authorities. The Ministry of Health was also notified, and meningitis surveillance was intensified across the Upper West, Northern, and Upper East regions. Health officials implemented control measures, including case management, contact tracing, community education on early identification of symptoms, and antimicrobial chemoprophylaxis for close contacts. As local measures led to increased awareness about meningitis, the number of reported cases increased. Health officials used community-based volunteers to identify possible meningitis cases and deaths. On February 12, at the request of the Ghanaian Ministry of Health, a team from the Ministry of Health and CDC joined local public health officials in the investigation.

A suspected meningitis case was defined as the occurrence of fever, neck stiffness, or other meningeal signs (e.g., headache, altered mental status, or bulging fontanelle in an infant) in a resident of northern Ghana. Patients with suspected meningitis who were evaluated at hospitals had a lumbar puncture performed for laboratory testing by Gram stain and culture or latex agglutination, where these were available. Probable cases were defined as the presence of at least one of the following in a patient with suspected meningitis: 1) turbid or cloudy CSF; 2) CSF white blood cell count >100/mm^3^; 3) CSF white blood cell count of 10–100/mm^3^ with either protein >100 mg/dl or glucose <40 mg/dl; or 4) an organism seen on Gram stain. Confirmed cases were defined as identification of a pathogen by real-time polymerase chain reaction (qPCR) in a patient with suspected or probable meningitis (CSF culture results were not included in the confirmed case definition because of lack of media and laboratory reagents required for pathogen growth and identification at health care facilities). Officials conducted active case finding, reviewed admission logbooks, and interviewed physicians treating patients. CSF specimens were also sent to the Tamale Public Health Laboratory, the reference laboratory for the three northern regions, for qPCR testing and serogrouping or serotyping of qPCR-positive specimens (*4*,*5*).

During December 8, 2015–April 3, 2016, a total of 1,006 suspected meningitis cases were reported, including 574 (57%) from the Upper West, 290 (29%) from the Northern, and 142 (14%) from the Upper East regions (Figure 1). During the first 10 weeks of the outbreak investigation (December 9, 2015–February 16, 2016), 432 suspected cases were identified among persons ranging in age from 1 month to 90 years; 44 (10%) met the probable case definition. Tamale laboratory received 286 CSF specimens for testing during December 9, 2015–February 16, 2016; among these, 133 (46.5%) were laboratory-confirmed (Figure 2). *N. meningitidis* was the most commonly detected pathogen among confirmed cases (n = 83, 62.4%), followed by *S. pneumoniae* (44, 33.1%) and *Haemophilus influenzae* (2, 1.5%). In four cases, more than one pathogen was detected: three had both *N. meningitidis* and *S. pneumoniae* and one had *S. pneumoniae* and *H. influenzae*. Among 103 confirmed cases with available outcome information, 8 (7.8%) were fatal. The case-fatality rate was higher among patients with pneumococcal meningitis (18.2%) than among those with meningococcal meningitis (3.1%) (p = 0.01).

**FIGURE 1:**
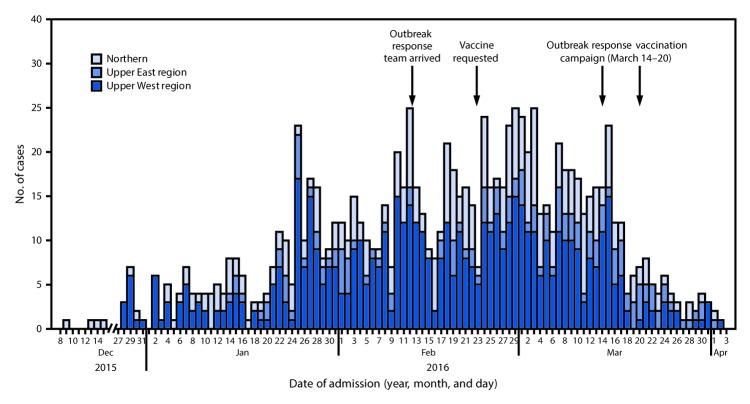
Suspected meningitis cases (N = 1,006), by date of admission and region and dates of vaccination campaigns with meningococcal polysaccharide ACW* vaccine — Northern Ghana, December 2015–April 2016 * *Neisseria meningitidis* serogroups A, C, and W.

**FIGURE 2:**
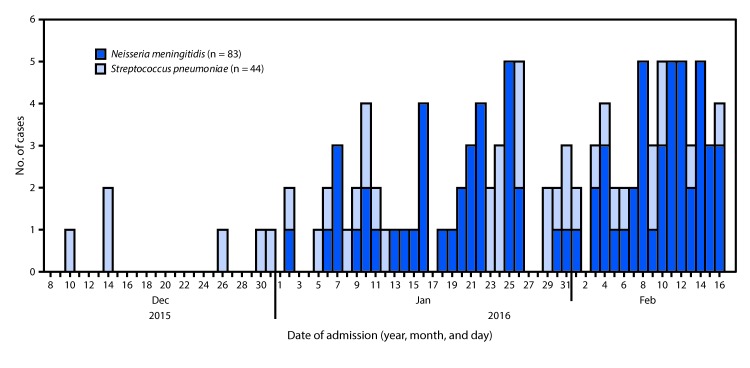
Laboratory-confirmed meningitis cases (N = 127),*^,†^ by date of admission and pathogen — Northern Ghana, December 9, 2015–February 16, 2016 * Among 432 suspected cases, 286 of which were laboratory tested, and 133 of which were confirmed. ^†^ Two confirmed *Haemophilus influenzae* cases and four cases with multiple pathogens excluded from figure.

Pneumococcal meningitis patients were significantly older (median age = 25 years, range = 3–72 years) than were meningococcal meningitis patients (median age = 15 years, range = 2–87 years); no significant differences were found by sex or geographic region (Table). Cases of pneumococcal meningitis were more prevalent early in the outbreak, whereas meningococcal meningitis cases predominated later in the outbreak (Figure 2). Among 83 meningococcal meningitis cases on which serogroups were examined, 82 (98.8%) were serogroup W; only one serogroup C case was detected. Among 37 serotyped pneumococcal meningitis cases, 20 (54%) were serotype 1, followed by serotype 23F (two cases, 5%), serotype 6A/6B (two, 5%), serogroup 18 (two, 5%); serotypes 3, 4, 5, 14, 19A, 19F and serogroup 12 accounted for one case each. CSF specimens from four patients with pneumococcal meningitis tested negative for all 21 serotypes/serogroups included in the qPCR assay and were probably caused by a different serotype. In the Upper West Region, the most affected region, meningococcal meningitis accounted for 42 (72.4%) of 58 confirmed cases (Table); in this region, three of 11 districts experienced rates above the epidemic threshold (10 suspected meningitis cases/100,000 population) during the week of February 7–13, 2016.

**TABLE:** Characteristics of confirmed meningitis cases (N = 127)* caused by *Streptococcus pneumoniae* and *Neisseria meningitidis*, by pathogen — northern Ghana, December 2015–February 2016

Characteristic	*Streptococcus pneumoniae* (n = 44)	*Neisseria meningitidis* (n = 83)	p-value
**Age (yrs), median (IQR)**	25 (15–42)	15 (8–28)	0.01
**Female, no. (%)**	21 (48.8)^†^	43 (52.4)^†^	0.70
**Region**			0.29
Northern region, no. (%)	21 (47.7)	32 (38.5)	
Upper West region, no. (%)	16 (36.4)	42 (50.6)	
Upper East region, no. (%)	7 (15.9)	9 (10.8)	
**Died, no. (%)**	6 (18.2)^§^	2 (3.1)^§^	0.01

**Abbreviation:** IQR = interquartile range.

* Four cases with >1 pathogen isolated and two cases with *Haemophilus influenzae* isolated were excluded.

^†^ Among patients whose sex was known: 43 with *S. pneumoniae* and 82 with *N. meningitidis*.

^§^ Among patients with outcome data available: 33 with *S. pneumoniae* and 65 with *N. meningitidis*.

On February 12, a team of four CDC epidemiologists and laboratorians joined local health authorities in Ghana to assist with the investigation. On February 23, based on the large number of confirmed meningitis cases caused by meningococcal serogroup W, Ghana Health Service, an autonomous executive agency responsible for implementation of national policies under the Ministry of Health, requested meningococcal serogroup W containing–vaccine from the International Coordinating Group for the most affected districts. On February 27, in conjunction with the Ghana Health Service, the International Coordinating Group released 160,000 doses of meningococcal polysaccharide ACW vaccine to the most affected districts in the Upper West Region. A mass outbreak response vaccination campaign was conducted during March 14–20, 2016, in collaboration with district officials, the national government, and the World Health Organization. By March 20, with assistance from the World Health Organization Ghana office, 135,679 persons aged 2–29 years had been vaccinated in three districts with coverage exceeding 98%.

## Discussion

The 2015–2016 meningitis outbreak in northern Ghana was caused by two main pathogens: *S. pneumoniae* predominated during the early weeks of the outbreak and *N. meningitidis* predominated during the latter. *S. pneumoniae* has been previously documented as the predominant pathogen at the beginning of meningitis outbreaks in Ghana, and it is not uncommon to identify cases of pneumococcal meningitis during meningococcal meningitis outbreaks (*6*). In this outbreak, persons with pneumococcal meningitis were older than those with meningococcal meningitis and were also older than persons with pneumococcal meningitis in outbreaks that occurred in Ghana before introduction of the 13-valent pneumococcal conjugate vaccine (PCV13) (*6*). PCV13 was introduced into Ghana’s national infant immunization program in 2012 as a 3-dose schedule at ages 6, 10, and 14 weeks^†^; children aged >4 years during this outbreak were not age-eligible to receive PCV13 when it was introduced. High coverage with PCV13 after 2012 likely resulted in the low pneumococcal infection rates observed in younger age groups. Among meningococcal meningitis cases, the age distribution was consistent with previous publications, indicating that persons aged 5–29 years are the primary carriers of *N. meningitidis* and are most affected during epidemics (*7*).

Almost all meningococcal meningitis cases in this outbreak were caused by serogroup W. Pneumococcal meningitis cases were caused by a number of different serotypes, predominantly serotype 1, which is one of the serotypes included in PCV13. This serotype, which is known to cause invasive pneumococcal infection (*8*), was associated with meningitis outbreaks in Africa before PCV introduction (*9*). This increase in serotype 1 pneumococcal meningitis in a country in which PCV13 has been introduced was surprising and might be related to several factors, including lack of a robust herd immunity (i.e., decrease in vaccine-type pneumococcus transmission because of childhood vaccination), reported low coverage the first year after introduction (43% in 2012),^§^ and potential waning of immunity to serotype 1 after the first year of life in the absence of a PCV13 booster dose.

The findings of this report are subject to at least four limitations. First, lack of laboratory reagents and supplies required for bacterial culture might have led to underdiagnosis or misidentification of the etiologic agent. Second, only 286 of 432 (66%) suspected meningitis cases had CSF specimens sent to the reference laboratory for qPCR testing. Third, outcome data were only available for a subset of patients with confirmed meningitis, and among those patients, it is possible that some might have died after case notification. Finally, some persons with severe bacterial meningitis might have died before seeking health care.

Rapid and coordinated response and collaboration among national and international partners led to prompt identification of the outbreak cause and implementation of control measures. The International Coordinating Group’s approval and provision of *N. meningitidis* serogroup W vaccine facilitated the mass reactive vaccination campaign, in which approximately 135,000 persons or 98% of the population aged 2–29 years in the most affected districts were vaccinated in less than 1 week. The International Coordinating Group does not maintain a stockpile of pneumococcal vaccine for outbreak response, because meningitis outbreaks in sub-Saharan Africa have been predominantly caused by *N. meningitidis*. Although pneumococcal mass vaccination could be used during pneumococcal meningitis outbreaks, the effectiveness of this approach in outbreak control needs to be better explored. It is still unclear if the same threshold used for meningococcal meningitis mass vaccination response can be used for pneumococcal outbreaks and what the optimal timeframe between outbreak onset and mass vaccination response should be for a pneumococcal vaccination campaign to have an impact in preventing further cases (*10*).

Outbreaks of bacterial meningitis are not uncommon in countries located in Africa’s meningitis belt. Rapid detection of the etiology of these outbreaks can lead to targeted public health interventions. Building and sustaining laboratory capacity in countries where meningitis outbreaks are common will be critical to ensure rapid and effective response to these outbreaks.

**Summary**

**What is already known about this topic?**

The introduction of serogroup A meningococcal conjugate vaccine (MenAfriVac) in Ghana in 2012 had a substantial impact on the periodic outbreaks of meningitis in the Northern Ghana. However, seasonal increases in bacterial meningitis continue to occur; the most prevalent etiologies are *Neisseria meningitidis*, *Streptococcus pneumoniae,* and *Haemophilus influenzae.*

**What is added by this report?**

During December 9, 2015–February 16, 2016, a total of 432 suspected meningitis cases were reported from three regions in northern Ghana. Among 286 cerebrospinal fluid specimens tested, 133 (46.5%) were positive, including 83 (62.4%) for *N. meningitidis* and 44 (33.1%) for *S. pneumoniae*. The predominant *N. meningitidis* serogroup was serogroup W (99%). Based on laboratory and epidemiologic data, 135,679 doses of meningococcal polysaccharide ACW vaccine were administered to the age groups most affected, resulting in substantial reduction in the number of meningitis cases.

**What are the implications for public health practice?**

Rapid identification of the etiologic agent in meningitis outbreaks is important for informing targeted public health interventions. Building and sustaining laboratory capacity in countries where meningitis outbreaks are common will be critical in ensuring rapid and effective response to these outbreaks.

## Footnotes

* The International Coordinating Group was established in 1997 following major outbreaks in Africa. It has four member agencies: the World Health Organization, Médecins sans Frontières, UNICEF, and the International Federation of Red Cross and Red Crescent Societies.

† International Vaccine Access Center (IVAC). http://view-hub.org/viz/#.

§ http://apps.who.int/immunization_monitoring/globalsummary/estimates?c=GHA.
